# The Knowledge, Attitude and Practice about Public Emergencies and the Response Capability of Residents in Shanghai after the Outbreak of Coronavirus Disease 2019 (COVID-19): A Cross-Sectional Study

**DOI:** 10.3390/ijerph18094814

**Published:** 2021-04-30

**Authors:** Jingting Lu, Xiaoqin Guo, Xiaoyu Han, Biao Deng, Qi Zhao, Genming Zhao, Na He

**Affiliations:** 1 Department of Epidemiology, School of Public Health, Fudan University, Shanghai 200032, China; 20211020032@fudan.edu.cn (J.L.); 15301020142@fudan.edu.cn (X.H.); 15301020137@fudan.edu.cn (B.D.); gmzhao@shmu.edu.cn (G.Z.); nhe@fudan.edu.cn (N.H.); 2 Key Laboratory of Public Health Safety (Ministry of Education), Shanghai 200032, China; 3 Songjiang Center for Disease Control and Prevention, Shanghai 201600, China; guoxiaoqin1102@163.com; 4 Department of Social Medicine, School of Public Health, Fudan University, Shanghai 200032, China; 5 NHC Key Laboratory of Health Technology Assessment, Shanghai 200032, China

**Keywords:** knowledge, attitude and practice, public health emergencies, emergency capability, residents

## Abstract

People’s knowledge, attitude and practice (KAP) are a part of the public’s emergency response capability and play an important role in controlling public health emergencies. This study aims to evaluate Shanghai residents’ KAP and the response ability regarding public health emergency events in China. An anonymous questionnaire investigation including demographics, socioeconomic characteristics and KAP was conducted through the online survey system from April 19 to April 30 2020. Of the 1243 people who completed the questionnaire, 1122 respondents (90.3%) had a good, positive attitude, while only 46.8% of participants had good knowledge, 46.2% performed well in the aspect of practice and 19.5% of residents had good response capability. It was found that men (OR:1.57,95% CI: 1.16–2.13), and people with 10 to 12 years or longer than 12 years of education (OR: 2.08,95% CI: 1.14–3.92; OR: 3.56,95% CI: 1.96–6.72) had the best public emergency response capability. Positive attitudes may be significantly associated with good practice (OR: 1.76, 95% CI: 1.18–2.64). Internet and television were the major media for people to acquire knowledge and skills. A lack of knowledge, poor perception and poor emergency response ability were found in Shanghai residents. Target intervention should be developed and implemented to improve the knowledge and skills of people for dealing with public emergency events.

## 1. Introduction

In today’s increasingly interconnected world, no community or country can be protected from public emergencies and disasters [1]. COVID-19 is a typical illustration of this. On January 30 2020, the World Health Organization (WHO) declared COVID-19 as a Public Health Emergency of International Concern [2] due to its rapid spread. Many studies reported that strengthening the construction of an individual’s or a household’s emergency response capability would greatly reduce the casualties of life or property. Proper household preparedness is one of the most effective measures to mitigate the effects of emergencies or disasters [3]. This public preparedness plays an important role, to some extent, in a city’s response capability and determines the progress and degree of a community’s recovery from an emergency [4,5].

Empirical research has suggested that household emergency preparedness can be affected by many factors such as knowledge, risk awareness and attitudes toward public health emergencies [1]. Some studies have also pointed out that people’s knowledge, attitude and practice (KAP), parts of their public health emergency response capability, play an important role in controlling the spread of an epidemic [6,7]. KAP affects people’s adherence to prevention and control measures, thus, eventually affecting the development or the implementation of preventive strategies and health promotion programs [6]. Assessing the public’s KAP helps us know their baseline information, identify the groups in the population with poor knowledge and correct people’s wrong ideas.

Until now, scholars all over the world have studied people’s KAP towards COVID-19 through self-designed questionnaires on the clinical symptoms or preventive measures, but few studies have comprehensively explored the KAP of participants towards public emergencies as a whole, let alone evaluated their response capability. This study aims to assess people’s KAP towards public emergencies after the pandemic of COVID-19, to identify subgroups with negative attitudes, poor practice and weak public emergency response capabilities, to analyze the main determinants of KAP and emergency response capability, and then provide information for addressing the public’s emergency response capacity.

## 2. Methods

### 2.1. Study Design

The cross-sectional study was conducted among residents of Shanghai from April 19 to April 30 when the lockdown of the city and across the whole country had been lifted, Shanghai’s epidemic prevention and control had reached a state of normal management, and there were no new local cases. Three communities of Songjiang district, which is located in the southwest of Shanghai with a permanent population of 1.76 million, were selected randomly.

### 2.2. Data Collection

A total of 1269 participants completed an online questionnaire through “Wenjuanxing” (a professional online questionnaire survey platform). A self-designed questionnaire, based on other published results, was used to collect data [8,9]. The questionnaire collected information on (1) demographic and socioeconomic characteristics (including gender, age, education, occupation and monthly income), (2) an individual’s knowledge of six categories of emergency events: typhoons, earthquakes, floods, fires, first aid, and infectious diseases, (3) an individual’s attitude, identified through three questions on emergency preparedness and health education, (4) an individual’s practice of wearing a mask and storing behavior of sixteen emergency supplies including water, food, flashlight and spare batteries, first aid kit, emergency medicine, radio, candles, matches or lighters, walkie talkie, a map, lifesaving whistle, thermometer, facemask, disinfectant, alcohol and hand sanitizer. The initial draft was sent to colleagues to ask for their opinions on improving the questionnaire. In order to control the quality of the questionnaire, participants were asked to complete the questionnaire within a specific range from 2.5 to 10 min. Each person could only submit the questionnaire once. Those with a completion time of less than 150 s were excluded during analysis. Twenty-six invalid questionnaires were eliminated and a total of 1243 participants’ questionnaires were analyzed. The total Cronbach’s alpha coefficient for the KAP questionnaire was 0.83, indicating that the internal consistency was acceptable [10].

### 2.3. Measurement

Participants’ knowledge of public emergencies was scored from 24 selected questions, with one point for each correct answer. The scores ranged from 0 to 24, with scores higher than 60% of all participants defined as good [11]. The total attitude score ranged from 0 to 3 and the participants who had at least one negative attitude were defined as negative. To determine participants’ practice, we used two questions: (1) whether they wore a mask or not, (2) and if they stored 16 necessary, emergency supplies at home. One point for each selection was awarded and the full score of the practice section was 17. A practice score higher than 60% of all participants was defined as good [11]. Participants with good knowledge, positive attitude and good practice at the same time were defined as having good emergency response capability.

### 2.4. Statistical Analysis

Frequencies of demographic and socioeconomic variables were described. A Chi-squared Test was used as a univariate analysis to compare the KAP level (good/poor) in different subgroups. Factors identified through the Chi-squared Test or based on our hypotheses were included in the multiple logistic regression analysis to identify the potential impact factors related to KAP. Univariate analysis and multiple logistic regression analysis were utilized for exploring the influencing factors for emergency response capability, as mentioned above.

All of the tests for significance were two-sided. The *p*-Values of univariate analysis <0.1 and multiple logistic regression analysis <0.05 were considered statistically significant. All analyses were conducted using the software R version 4.0.2.

## 3. Results

Of the total participants, 488 (39.9%) were men and 755 (60.7%) were women. Most of the participants (33.9%) were between the ages of 31 and 40. There were 796 (64.0%) participants who had received more than 13 years of education. Nearly a third of respondents (30.4%) were technical professionals such as doctors, teachers and researchers. In terms of monthly income, almost half of the residents (47.5%) were in the group earning 2000–4999 CNY (Table 1).

The median of the knowledge, attitude and practice scores were 18.00 [16.00,20.00], 3.00 [3.00,3.00] and 12.00 [9.00,15.00], respectively. As shown in Table 2, there were significant differences between age groups (*p* = 0.04), levels of education (*p* < 0.01), occupations (*p* < 0.01) and monthly income (*p* < 0.01). Multiple logistic regression analysis showed that men were 46% more likely to achieve a good knowledge score than women (OR: 1.46, 95% CI: 1.13–1.90). Compared with participants who had received less than 9 years of education, people with more years of education tended to perform better in the aspect of knowledge (10–12 years of education: OR: 1.64, 95% CI: 1.05–2.57; ≥13 years of education: OR: 2.64, 95% CI: 1.69–4.16). The group of professional and technical staff had a significant association with good knowledge when compared to the group of workers or farmers (OR:3.36, 95% CI: 2.21–5.13), and the participants whose monthly income was more than 5000 CNY were more likely to have a good knowledge of public emergencies compared with those who earned less than 1999 CNY (5000–9999 CNY: OR: 2.17, 95% CI: 1.05–4.76; ≥10,000 CNY: OR: 2.49, 95% CI: 1.08–6.03) (Table 3).

The majority of participants (90.3%) held a positive attitude towards making preparations for public emergencies. The Chi-squared test showed that attitudes were quite different among participants with different education levels (*p* < 0.01), occupations (*p* = 0.02) and monthly income (*p* < 0.01) (Table 2). It was found that residents who received more than 13 years of education were more likely to have a positive perception of emergencies than those who had received less than 9 years of education (OR: 2.58, 95% CI: 1.32–5.04); participants with a monthly income from 2000 to 4999 and 5000 to 9999 CNY showed a more positive attitude than those with a monthly income less than 1999 CNY (2000–4999 CNY: OR: 2.83, 95% CI: 1.30–5.93; 5000–9999 CNY: OR: 2.38, 95% CI: 1.01–5.44) (Table 3).

In terms of participants’ practice, capability significantly differed across all social and demographic variables. Multiple logistic regression analysis showed that men were 50% more likely to have better practice than women (OR: 1.50, 95% CI: 1.17–1.91). It was also found that positive attitudes may be significantly associated with good practice (OR: 1.76, 95% CI: 1.18–2.64) (Table 3).

**Figure 1:**
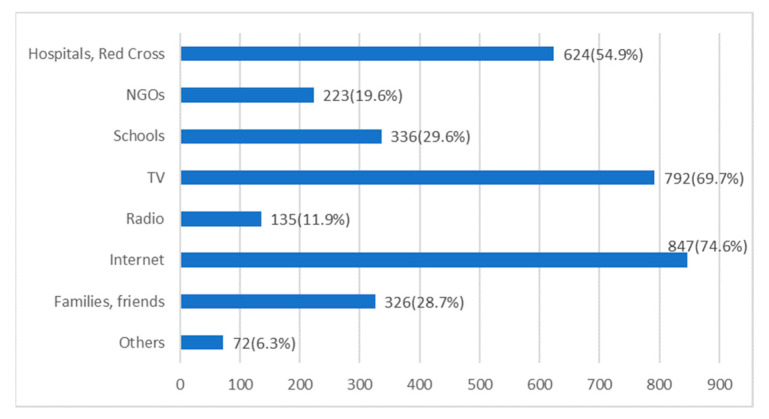
Ways to acquire knowledge and skills towards public emergencies.

Only 243 (19.5%) residents were considered as having good emergency capability. Univariate analysis showed that people’s response capability was quite different in the groups of gender (*p* = 0.02) and years of education (*p* < 0.01). This was also different in some subgroups of age (31–40: *p* = 0.06; ≥51: *p* = 0.06), occupation (professional and technical staff: *p* = 0.01) and monthly income (5000–9999 CNY: *p* = 0.04; ≥10000 CNY: *p* = 0.04) (Table 4). Multiple logistic regression analysis was performed, and the result suggested that men were 57% more likely to have good emergency capability (OR: 1.57, 95% CI: 1.16–2.13). Both groups of participants who had received 10–12 years of education (OR: 2.08, 95% CI: 1.14–3.92) or more than 13 years of education (OR: 3.56, 95% CI: 1.96–6.72) tended to have better emergency capability compared with those who had received less than 9 years of education (Table 4). In addition, how respondents acquired their skills and knowledge of public emergencies was also investivated, and it was found that the internet and television were the major media used by people to acquire knowledge and skills (Figure 1).

## 4. Discussion

From our study, we found that more than half of respondents had poor knowledge or practice. When it comes to a good emergency response capability, the number of people who had good capability was fewer. Only one in five residents had good response capability.

Since the outbreak of COVID-19, the public has received a great deal of health education about the disease from all directions and through multiple channels. Maybe this is the reason why participants got good results for their knowledge of and practice in the aspect of infectious diseases, and why most held a very positive attitude towards acquiring knowledge, skills and reserving materials for unknown or upcoming disasters, but for other public emergencies, such as typhoons and earthquakes, their performance was relatively poor. This informed us that the government or social media could strengthen the popularization of other public health emergency response knowledge and measures appropriately while popularizing the knowledge of COVID-19, so as to improve people’s capability to respond to public emergencies comprehensively.

Our analysis suggested that, for people who had received more than 9 years of education and especially more than 13 years, this may be the protective factor for residents’ knowledge and attitude, which was in line with our judgements. People with higher education may have more access to information from various sources, thus enabling them to possess more knowledge about public emergencies and to be more positive [12].

Since the group of professional and technical staff consisted of teachers, doctors and researchers who had a higher level of education and usually paid more attention to COVID-19 or other public emergencies, this may be why they would get a higher score for knowledge than farmers and workers in general [12,13].

The results of multiple logistic regression showed that a monthly income of 5000–9999 and ≥10,000 CNY could lead to a resident’s better knowledge, while people in the income group of 2000–4999 and 5000–9999 CNY held a more positive attitude than those who earned less than 1999 CNY every month. This may be because a person’s economic level is linked to their education level. Researchers found that people with higher education were more likely to gain better employment and consequently have a higher monthly income [14]. Besides, people with higher income could afford to spend more money on personal protective equipment such as masks [12], which indirectly reflects their more positive attitude. These two reasons may explain the results above. This also indicates that economic factors may be important in residents’ KAP. A more detailed analysis should be conducted in the future to explore the influence level and the trend between monthly income and residents’ KAP.

Moreover, the results showed that being female was a common factor that may lead to participants’ poor knowledge and practice; this could be caused by women’s personality, mental stress or showing no interest in emergency preparedness [15].

Similar to Harapan’s study [16], the results indicated that people who had good attitudes were more likely to have good practice for emergency capability.

The result of the evaluation of participants’ response capability was not optimistic as only 19.5% of residents had a relatively good emergency response capability. Men were more likely to prepare well for unknown emergencies than women, which was a similar finding to previous studies [17,18,19] and the same result as for participants’ good practice above. In the traditional view of people from China, especially those who are middle-aged or elderly, men should take on more family responsibilities, so they may pay more attention to relevant information or knowledge [9,20]. In the same way as for participants’ knowledge, education level played an important role in people’s public emergency response capability. Therefore, in the future, health education among the female, low-educated or low-income population needs to be more focused.

Interestingly, some researchers also found that people who were 55 years of age or older and had higher incomes tended to have better response capability [17,18], while our analysis did not achieve such a conclusion. There may be many reasons for this: on the one hand, content on infectious diseases accounted for a large proportion of the questionnaire, and as the short-term effect still existed where people had just experienced COVID-19, the distinction in population between different age groups may not have been so obvious. On the other hand, there were few participants with a monthly income less than 1999 CNY or older than 50 years old, and their distribution was spindle-shaped, so it was difficult to find a positive result when selecting these two groups as the reference.

Currently, TV and the internet are still the main ways for people to acquire knowledge. However, these may sometimes mislead people, since there is some fake and unproven information on the internet [21,22,23]. While our participants have different education levels or abilities to distinguish true or false information, the related government departments, the Red Cross, hospitals and even schools should take more responsibility in conducting health education and propagate and teach correct emergency knowledge and skills. The mass media should also pay attention to the direction of public opinion during public emergencies and, when delivering scientific and reliable information in time, they should not hype some inappropriate remarks or exaggerate the situation, so as not to have a negative impact on the prevention and control of the emergency [24].

To the best of our knowledge, this study is one of few studies to explore the KAP of residents towards public emergencies after the outbreak of COVID-19 and to evaluate the response capability on the basis of KAP, yet limitations still exist.

First, because of the outbreak of COVID-19, the cross-sectional study was conducted through the internet and the data were collected from the self-reported questionnaires online without any guidance or supervision of investigators. Therefore, there may be some inevitable mistakes in collecting data. For example, some people may have deliberately lied about certain behaviors because they were more likely to be recognized. Second, all of the participants included in the study were living in Shanghai, so the results of the study cannot represent the whole of China. Finally, participants’ practice score or their response capability may be overestimated, since the practice section of the questionnaire included five virus prevention supplies; in addition, we did not study participants’ response capability before COVID-19, so we cannot clearly compare the difference caused by the epidemic. In the future, a further study should be conducted to learn more about the change in people’s response ability towards public emergencies after COVID-19.

## 5. Conclusions

In conclusion, our study surveyed people’s KAP towards public emergencies after the outbreak of COVID-19 and evaluated their emergency response ability. The results indicated that there were still some gaps between people’s response ability and KAP towards public emergencies. In the future, a more scientific, complete and detailed emergency ability education system needs to be established. Improving emergency response ability is not only the function of the government but individuals should also pay attention to improving their own capabilities. The government can utilize the internet, mass media, WeChat official accounts and so on to carry out extensive publicity and education, while individuals should be encouraged to take part in relevant health promotion programs to strengthen their emergency response capability, so as to make preparations for unknown public emergencies and, ultimately, reduce losses.

## Figures and Tables

**Table 1:** Social and demographic characteristics of the study participants.

Characteristics	N	%
Gender		
Male	488	39.3
Female	755	60.7
Age (years)		
≤25	131	10.5
26–30	152	12.2
31–40	421	33.9
41–50	375	30.2
≥51	164	13.2
Education (years)		
≤9	218	17.5
10–12	229	18.4
≥13	796	64.0
Occupation		
Workers or farmers	186	15.0
Professional and technical staff	378	30.4
Service staff	237	19.1
Government employees	170	13.7
Others	272	21.9
Monthly income (CNY)		
≤1999	51	4.1
2000–4999	590	47.5
5000–9999	500	40.2
≥10,000	102	8.2

**Table 2:** Differences in knowledge, attitude and practice of Shanghai residents by demographics (N = 1243).

Characteristics	Knowledge			Attitude			Practice		
	Good	Poor	*p*	Positive	Negative	*p*	Good	Poor	*p*
Overall	N (%)	N (%)		N (%)	N (%)		N (%)	N (%)	
Gender									
Male	235 (48.2)	253 (51.8)	0.48	431 (88.3)	57 (11.7)	0.08 *	257 (52.7)	231 (47.3)	<0.01 **
Female	347 (46.0)	408 (54.0)		691 (91.5)	64 (8.5)		317 (42.0)	438 (58.0)	
Age (years)									
≤25	69 (52.7)	62 (47.3)	0.04 **	121 (92.4)	10 (7.6)	0.17	66 (50.4)	65 (49.6)	0.09 *
26–30	75 (49.3)	77 (50.7)		135 (88.8)	17 (11.2)		63 (41.4)	89 (58.6)	
31–40	205 (48.7)	216 (51.3)		385 (91.4)	36 (8.6)		180 (42.8)	241 (57.2)	
41–50	173 (46.1)	202 (53.9)		341 (90.9)	34 (9.1)		177 (47.2)	198 (52.8)	
≥51	60 (36.6)	104 (63.4)		140 (85.4)	24 (14.6)		88 (53.7)	76 (46.3)	
Education (years)									
≤9	46 (21.1)	172 (78.9)	<0.01 **	186 (85.3)	32 (14.7)	<0.01 **	116 (53.2)	102 (46.8)	<0.01 **
10–12	83 (36.2)	146 (63.8)		190 (83.0)	39 (17.0)		121 (52.8)	108 (47.2)	
≥13	453 (56.9)	343 (43.1)		746 (93.7)	50 (6.3)		337 (42.3)	459 (57.7)	
Occupation									
Workers or farmers	60 (32.3)	126 (67.7)	<0.01 **	168 (90.3)	18 (9.7)	0.02 **	84 (45.2)	102 (54.8)	<0.01 **
Professional and technical staff	262 (69.3)	116 (30.7)		354 (93.7)	24 (6.3)		147 (38.9)	231 (61.1)	
Service staff	73 (30.8)	164 (69.2)		208 (87.8)	29 (12.2)		126 (53.2)	111 (46.8)	
Government employees	77 (45.3)	93 (54.7)		157 (92.4)	13 (7.6)		81 (47.6)	89 (52.4)	
Others	110 (40.4)	162 (59.6)		235 (86.4)	37 (13.6)		136 (50.0)	136 (50.0)	
Monthly income (CNY)									
≤1999	12 (23.5)	39 (76.5)	<0.01 **	38 (74.5)	13 (25.5)	<0.01 **	30 (58.8)	21 (41.2)	0.01 **
2000–4999	217 (36.8)	373 (63.2)		532 (90.2)	58 (9.8)		293 (49.7)	297 (50.3)	
5000–9999	289 (57.8)	211 (42.2)		461 (92.2)	39 (7.8)		208 (41.6)	292 (58.4)	
≥10,000	64 (62.7)	38 (37.3)		91 (89.2)	11 (10.8)		43 (42.2)	59 (57.8)	

* *p* < 0.1, ** *p* < 0.05.

**Table 3:** Multiple logistic regression on factors significantly associated with good KAP towards public emergency.

Characteristics	Good Knowledge		Positive Attitude		Good Practice	
	OR (95% CI)	*p*	OR (95% CI)	*p*	OR (95% CI)	*p*
Gender						
Female	1.00	-	1.00	-	1.00	-
Male	1.46 (1.13–1.90)	<0.01 **	0.72 (0.48–1.08)	0.11	1.50 (1.17–1.91)	<0.01 *
Age (years)						
≤25	1.00	-	1.00	-	1.00	-
26–30	0.87 (0.52–1.44)	0.59	0.54 (0.22–1.26)	0.16	0.75 (0.46–1.22)	0.24
31–40	0.94 (0.61–1.46)	0.78	0.82 (0.36–1.73)	0.61	0.76 (0.50–1.15)	0.19
41–50	1.12 (0.71–1.78)	0.62	1.04 (0.44–2.25)	0.93	0.86 (0.56–1.33)	0.5
≥51	0.89 (0.51–1.54)	0.67	0.94 (0.39–2.18)	0.89	0.95 (0.57–1.58)	0.84
Education (years)						
≤9	1.00	-	1.00	-	1.00	-
10–12	1.64 (1.05–2.57)	0.03 **	0.77 (0.43–1.34)	0.35	1.07 (0.72–1.60)	0.72
≥13	2.64 (1.69–4.16)	<0.01 **	2.58 (1.32–5.04)	0.01 **	0.84 (0.56–1.27)	0.41
Occupation						
Workers or farmers	1.00	-	1.00	-	1.00	-
Professional and technical staff	3.36 (2.21–5.13)	<0.01 **	0.81 (0.38–1.70)	0.59	0.98 (0.65–1.47)	0.93
Service staff	1.01 (0.65–1.55)	0.98	0.85 (0.44–1.60)	0.61	1.36 (0.92–2.02)	0.13
Government employees	1.20 (0.76–1.90)	0.44	0.91 (0.41–2.05)	0.82	1.31 (0.84–2.04)	0.24
Others	1.49 (0.98–2.26)	0.06	0.60 (0.31–1.12)	0.11	1.31 (0.89–1.95)	0.17
Monthly income (CNY)						
≤1999	1.00	-	1.00	-	1.00	-
2000–4999	1.47 (0.73–3.16)	0.29	2.83 (1.30–5.93)	0.01 **	0.68 (0.36–1.25)	0.22
5000–9999	2.17 (1.05–4.76)	0.04 **	2.38 (1.01–5.44)	0.04 **	0.55 (0.28–1.05)	0.07
≥10,000	2.49 (1.08–6.03)	0.04 **	1.40 (0.50–3.95)	0.52	0.58 (0.27–1.24)	0.16
knowledge	-	-				
Poor			1.00	-	1.00	-
Good			1.4 (0.92–2.17)	0.12	1.00 (0.78–1.28)	0.99
Attitude	-	-	-	-		
Negative					1.00	-
Positive					1.76 (1.18–2.64)	<0.01 *

OR: odds ratio, CI: confidence interval. * *p* < 0.1, ** *p* < 0.05.

**Table 4:** Univariate and multiple logistic regression on factors associated with good response capability towards public emergencies.

Characteristics	Capability		Univariate		Multivariate	
	Good (N (%))	Poor (N (%))	OR (90% CI)	*p*	aOR (95% CI)	*p*
Gender						
Female	111 (22.7)	377 (77.3)	1.00	-	1.00	-
Male	132 (17.5)	623 (82.5)	1.39 (1.10–1.76)	0.02 **	1.57 (1.16–2.13)	<0.01 **
Age (years)						
≤25	34 (26.0)	97 (74.0)	1.00	-	1.00	-
26–30	28 (18.4)	124 (81.6)	0.64 (0.40–1.03)	0.13	0.61 (0.34–1.10)	0.10
31–40	77 (18.3)	344 (81.7)	0.64 (0.44–0.95)	0.06 *	0.63 (0.39–1.04)	0.06
41–50	76 (20.3)	299 (79.7)	0.73 (0.49–1.08)	0.18	0.93 (0.56–1.56)	0.77
≥51	28 (17.1)	136 (82.9)	0.59 (0.36–0.94)	0.06 *	0.90 (0.48–1.69)	0.74
Education (years)						
≤9	18 (8.3)	200 (91.7)	1.00	-	1.00	-
10–12	38 (16.6)	191 (83.4)	2.21 (1.35–3.69)	0.01 **	2.08 (1.14–3.92)	0.02 **
≥13	187 (23.5)	609 (76.5)	3.41 (2.27–5.35)	<0.01 **	3.56 (1.96–6.72)	<0.01 **
Occupation						
Workers or farmers	27 (14.5)	159 (85.5)	1.00	-	1.00	-
Professional and technical staff	93 (24.6)	285 (75.4)	1.92 (1.31–2.88)	0.01 **	1.36 (0.82–2.30)	0.24
Service staff	37 (15.6)	200 (84.4)	1.09 (0.70–1.72)	0.76	1.13 (0.65–1.98)	0.67
State employees	33 (19.4)	137 (80.6)	1.42 (0.89–2.27)	0.22	1.03 (0.58–1.85)	0.92
Others	53 (19.5)	219 (80.5)	1.43 (0.94–2.20)	0.17	1.39 (0.82–2.39)	0.22
Monthly income (CNY)						
≤1999	5 (9.8)	46 (90.2)	1.00	-	1.00	-
2000–4999	100 (16.9)	490 (83.1)	1.88 (0.91–4.55)	0.19	1.74 (0.69–5.34)	0.28
5000–9999	113 (22.6)	387 (77.4)	2.69 (1.30–6.50)	0.04 **	1.84 (0.71–5.75)	0.24
≥10,000	25 (24.5)	77 (75.5)	2.99 (1.33–7.63)	0.04 **	1.94 (0.67–6.57)	0.25

OR: odds ratio, CI: confidence interval. * *p* < 0.1, ** *p* < 0.05.

## Data Availability

The data presented in this study are available on request from the corresponding author.
